# Issues and Prospects of Current Endoscopic Treatment Strategy for Superficial Non-Ampullary Duodenal Epithelial Tumors

**DOI:** 10.3390/curroncol29100537

**Published:** 2022-09-22

**Authors:** Tetsuya Suwa, Masao Yoshida, Hiroyuki Ono

**Affiliations:** Division of Endoscopy, Shizuoka Cancer Center, Shizuoka 411-8777, Japan

**Keywords:** superficial non-ampullary duodenal epithelial tumor, conventional endoscopic mucosal resection, endoscopic submucosal dissection, cold snare polypectomy, underwater endoscopic mucosal resection, endoscopic full-thickness resection, laparoscopic and endoscopic cooperative surgery for duodenal tumors, treatment strategy

## Abstract

An increasing number of duodenal tumors are being diagnosed over the years, leading to increased confusion regarding the choice of treatment options. Small-to-large tumors and histological types vary from adenoma to carcinoma, and treatment methods may need to be selected according to lesion characteristics. Because of its anatomic characteristics, complications are more likely to occur in the duodenum than in other gastrointestinal organs. Several reports have described the outcomes of conventional endoscopic mucosal resection, endoscopic submucosal dissection, cold snare polypectomy, underwater endoscopic mucosal resection, endoscopic full-thickness resection, and laparoscopic and endoscopic cooperative surgery for duodenal tumors. However, even in the guidelines set out by various countries, only the treatment methods are listed, and no clear treatment strategies are provided. Although there are few reports with a sufficiently high level of evidence, considering the currently available treatment options is essential. In this report, we reviewed previous reports on each treatment strategy, discussed the current issues and prospects, and proposed the best possible treatment strategy.

## 1. Introduction

As the opportunities to diagnose superficial non-ampullary duodenal epithelial tumors (SNADETs) have been increasing, healthcare personnel more often encounter a variety of SNADETs with respect to tumor size and histological grade of atypia [1]. However, a standardized strategy for the endoscopic treatment of SNADETs has not yet been established, although several treatment guidelines for SNADETs have been published by major associations [2,3,4]. Thus, treatment strategies composed of conventional endoscopic mucosal resection (cEMR), cold snare polypectomy (CSP), underwater endoscopic mucosal resection (UEMR), endoscopic submucosal dissection (ESD), endoscopic full-thickness resection (EFTR), and laparoscopic and endoscopic cooperative surgery (LECS) are performed depending on the medical resources of each institution and clinician preferences. The duodenum has the following anatomical disadvantages: a thin proper muscular layer, poor operability of the endoscope, the retroperitoneal organ close to the pancreas, and exposure to bile and pancreatic juice, which cause more severe and fatal adverse events (AEs) after endoscopic treatment. To avoid AEs related to endoscopic treatment, CSP and UEMR have been widely used as minimally invasive endoscopic treatments. However, the indications for the various endoscopic treatment methods, including other endoscopic treatments, such as cEMR and ESD, are unclear because of insufficient evidence regarding their advantages and disadvantages. This article reviews previous reports on the endoscopic treatment for SNADETs by referring to our results of CSP and UEMR, and discusses issues and prospects. Although there are not many high-quality reports in the field of SNADET treatment, we searched databases, including PubMed, and cited previous reports with a higher level of evidence as much as possible.

## 2. Overview of Clinical Practice Guidelines

In the guidelines of the American Society for Gastrointestinal Endoscopy [2], the indications for the various endoscopic resection methods are not mentioned. However, the guidelines state that the general approach should be similar to that for colon polyps, particularly those on the right side of the colon with thin walls, such as the duodenum. The guidelines also mention that endoscopic procedures for SNADETs have higher AE rates than for lesions of the same size in the colon, and this is also true in duodenal ESD.

The European Society of Gastrointestinal Endoscopy (ESGE) guidelines [3] recommend CSP for adenomas < 6 mm in size, and that larger SNADETs should be resected using cEMR as the standard endoscopic procedure. Additionally, ESD should be performed by experts alone, without any recommendation of the tumor size for ESD. The ESGE guidelines also state that ESD should be performed in cases of suspected invasive cancer in the shallow submucosal layer or positive non-lifting signs due to fibrosis, considering the high AE rate. Although UEMR and piecemeal CSP have been described as alternative treatments for cEMR, their indications have not been clearly mentioned.

The Japanese guidelines [4] divided SNADETs into three categories based on the tumor size of ≤10 mm, 11–20 mm, and >20 mm, and outlined observation, CSP, EMR (including cEMR and UEMR), ESD, and LECS as other relevant treatments. However, the indications and recommendations for each method were unstated, and ESD was weakly recommended (for experts alone), as in the ESGE guidelines.

## 3. Review of Endoscopic Treatment for SNADETs

### 3.1. Conventional Endoscopic Mucosal Resection (cEMR)

Initially, cEMR was the only option for the endoscopic treatment of SNADETs and was applied for any SNADET size. Presently, cEMR is commonly performed because many endoscopists have accumulated sufficient experience of colorectal polyps. Before the development of ESD and other invasive treatments, cEMR was performed on relatively large lesions for which such treatments are now indicated; however, according to the data from that period, higher AE rates were observed [5]. However, considering recent comparisons with other treatment options and improvements in suturing techniques after resection, cEMR is considered a relatively safe procedure. Furthermore, when discussing complications, we recently discussed them separately as ESD or otherwise [6].

Nonaka et al. [7] reported less favorable outcomes of cEMR for 113 SNADETs; the *en-bloc* resection rate was 63% (71/113) and the R0 resection rate was 34% (38/113). In the same report, the *en-bloc* resection rate decreased as lesion size increased: 69% (68/99) for lesions < 20 mm, and 21% (3/14) for lesions > 20 mm. Perioperative perforation did not occur, and delayed bleeding occurred in 12% (14/113) of patients. It is important to note that residual recurrence was not observed during the long follow-up period (median (range): 51 (12–163) months), although the piecemeal resection rate was 37% in this report.

Yahagi et al. [8] reported excellent outcomes of cEMR for 146 SNADETs and ESD for 174 SNADETs; the *en-bloc* resection and R0 resection rates were 95.2% vs. 98.3% (no significant difference) and 82.2% vs. 85.1% (no significant difference), respectively, indicating that cEMR might be comparable to ESD. Furthermore, the rate of AEs showed a delayed bleeding rate of 1.4% vs. 5.2% (*p* = 0.072), whereas the perforation rate was significantly lower with cEMR (0.68% vs. 15.5%, *p* < 0.001). Interestingly, the sample size was small, but this report also showed that cEMR achieved 100% *en-bloc* resection for lesions 21–30 mm in size, although 33.3% of lesions were >30 mm. Although the size cutoffs were different, these results suggest that a larger size worsened the treatment outcome.

Additionally, Kato et al. [6] reported the largest, high-volume, multicenter study. Although the *en-bloc* resection and R0 resection rates of cEMR were inferior to those of ESD (86.8% vs. 94.8% and 61.2% vs. 78.7%, respectively), the rate of AEs (delayed bleeding, delayed perforation, and conversion to emergency surgery rates) of cEMR was significantly lower than that of ESD (2.6% vs. 4.7%, 0.2% vs. 2.3%, and 0.07% vs. 2.5%, respectively). The authors also indicated that, compared with the ESD group, the AE rates were lower for SNADETs < 30 mm and higher for SNADETs > 30 mm in the non-ESD group.

Considering the above results, cEMR could reduce the AE rate, although the *en-bloc* resection and R0 resection rates were lower than those of ESD. Regarding indications, it was possible to resect SNADETs < 20 mm safely and reliably with the necessary clinical experience. However, despite the small sample size in previous reports, cEMR would be unacceptable for lesions > 30 mm because the *en-bloc* resection rate was significantly lower and the complication rates were higher than those of ESD, in reference to a previous report. Although piecemeal EMR tends to increase the recurrence rate [8], Nonaka et al. [7] described no recurrence in a population with a high piecemeal EMR rate. In piecemeal cases, high-quality techniques are required so that no endoscopic residue remains. Moreover, 20–30 mm lesions might be acceptable for piecemeal resection, considering the low risk of residual recurrence even without *en-bloc* resection.

### 3.2. Endoscopic Submucosal Dissection (ESD)

ESD is a standard procedure for early-stage gastrointestinal cancers > 20 mm, except for the duodenum, because of a high AE rate. The high complication rate was considered to be due to the following duodenal anatomical characteristics: the very thin duodenal wall, the retroperitoneal organ near the pancreas, exposure to bile and pancreatic juice, and poor endoscopic maneuverability. However, several high-volume centers have reported remarkable ESD outcomes, and previous reports should be reviewed considering the accumulated experience and improved prophylactic suturing techniques.

Yahagi et al. [9] reported excellent outcomes with ESD performed for 174 SNADETs; the average lesion size was 27.4 ± 16.1 mm, the *en-bloc* resection rate was 98.3%, and the R0 resection rate was 85.1%. However, the delayed bleeding and perforation rates were relatively high (5.2% and 15.5%, respectively), and the AE rates remained higher than those of other endoscopic resection methods.

Regarding size, Hoteya et al. [10] compared the outcomes of ESD for 49 large lesions (>20 mm) and 25 small lesions (≤20 mm) with those of cEMR for 55 lesions of any size. The *en-bloc* resection rates were 98% vs. 100% vs. 78.2%, respectively, and were significantly higher in the ESD group, regardless of size. In contrast, delayed bleeding and perforation rates were 14.3% and 2.0% vs. 16.0% and 0% vs. 7.3% and 0%, respectively. Notably, the authors suggested that delayed bleeding and perforation could be improved if prophylactic suturing was performed in each procedure (5.9% and 0% vs. 0% and 0% vs. 4.2% and 0%, respectively).

In a report by Kato et al. [6], the incidence of AEs was higher in ESD than in non-ESD for lesions < 30 mm, but higher in non-ESD than in ESD when the lesion size was ≥30 mm, indicating that a large lesion might be a risk factor for complications, which was related to the possibility of complete suturing. To reduce complication rates, new devices and strategies, such as the over-the-scope clip (OTSC) and string clip suturing methods, have been reported, and further innovations are desired.

In conclusion, ESD is currently an option for treating SNADETs > 20 mm, and the procedure should be performed only by experts in high-volume centers. Poor scope maneuverability is a concern in ESD, and piecemeal EMR, EFTR, and LECS may be considered when the completion of the procedure is considered difficult.

### 3.3. Cold Snare Polypectomy (CSP)

We reported the treatment outcomes of CSP in 47 patients with 53 lesions who underwent CSP for SNADETs at the Shizuoka Cancer Center from January 2015 to July 2020; details are shown in Table 1 [11].

Okimoto et al. [12] reported the outcomes of CSP for 47 lesions with a median endoscopic size of 4 mm. The *en-bloc* and R0 resection rates were 97.8% and 70.3%, respectively. In the long-term outcomes of patients followed up for more than 1 year, the residual recurrence rate was 2.7%, which could be treated with repeat endoscopic procedures (CSP and cold forceps polypectomy).

Considering the results of CSP for colorectal polyps, the R0 resection rate was significantly higher in the <10 mm group than in the ≥10 mm group (73% vs. 54%) [13], and it might be impossible to resect lesions with negative margins, including the muscularis mucosa and submucosal layer [14]; CSP should not be used to resect lesions suspicious for carcinoma.

Based on the above results, CSP could be indicated for adenomas of ≤10 mm, although the ESGE guidelines indicated CSP for adenomas of <6 mm. However, CSP is associated with several challenges when selecting the treatment option. First, the preoperative diagnostic accuracy for SNADETs is not suitable: in studies, the diagnostic capability of Vienna 4 or higher using white-light imaging and narrow-band imaging was insufficient [15,16]. Furthermore, the preoperative biopsy was not useful [17]. Second, refraining from treatment with a short interval follow-up might be an option for small lesions < 10 mm in size, and the Japanese guidelines list follow-up as one of the treatment strategies. However, Okada et al. [18] reported that approximately 25.9% (11/43) of low-grade adenomas progressed to high-grade dysplasia or noninvasive carcinomas over 6 months, and it was reasonable to endoscopically treat the small lesions, while minimally invasive modalities with fewer complications such as CSP could be adapted. Finally, the distinction between CSP and other endoscopic resection techniques, such as cEMR and UEMR, is unclear. An important characteristic of CSP is non-electrocautery resection, which involves mechanical strangulation. While the burn effect obtained with the electrocautery resection has the potential advantage of preventing residual tumor, a disadvantage is the possibility of increasing the risk of delayed bleeding due to microvascular damage. Hamada et al. [19] reported the safety of CSP in patients with multiple duodenal tumors in familial adenomatous polyposis cases. In this report, no complications, including delayed bleeding, were observed, even without prophylactic clipping after CSP for multiple lesions, which suggests that prophylactic clipping could be passed to prevent AEs in CSP.

Although CSP has the advantages of being a minimally invasive treatment with a low risk of complications and a simple procedure not requiring submucosal injection or electrocautery, a careful indication of the lesion is required, and it is essential to improve the diagnostic accuracy rate in the preoperative evaluation.

In summary, CSP should be indicated only for duodenal adenomas ≤10 mm in size. Although follow-up is important, we recommend aggressively resecting small lesions (≤10 mm) with minimally invasive methods such as CSP because the natural history of SNADETs has not been elucidated.

### 3.4. Underwater Endoscopic Mucosal Resection (UEMR)

We reported the treatment outcomes of UEMR for 65 patients with 54 lesions who underwent UEMR for SNADET at the Shizuoka Cancer Center from January 2015 to July 2020; details are shown in Table 2 [11].

Yamasaki et al. [20] reported the safety and efficacy of UEMR in a multicenter prospective study of 155 patients with 166 lesions; the mean endoscopic tumor size was 10.0 ± 4.1 mm, the location (1st/2nd/3rd) was 10/151/5, the macroscopic type in the Paris classification (0-Is/0-IIa/0-IIc) was 18/106/42, the mean procedure time was 5.4 ± 4.3 min, the *en-bloc* resection rate was 89.8%, the R0 resection rate was 66.9%, the prophylactic clipping rate was 99.4%, the intraoperative bleeding and perforation rates were 2.4% and 0%, and the delayed bleeding and perforation rates were 1.2% and 0%. Of the 144 lesions followed up to 12 months after UEMR, residual recurrence was observed in 4 lesions (2.8%), all of which could be treated with re-endoscopic resection (UEMR: 2, EMR with hot biopsy ablation: 1, cold polypectomy with argon plasma coagulation: 1).

Although the R0 resection rate was low, similar to our data, no perforation that might have led to additional surgical resection was observed, and all residual recurrent lesions could be endoscopically treated, suggesting that UEMR was adequately indicated for lesions measuring ≤20 mm.

Table 3 summarizes the treatment outcomes of UEMR reported by size (<10 mm, 10–20 mm, >20 mm) in detail. The R0 resection rate was approximately 60–80%, but the *en-bloc* resection rate was high (≥90%), and the complication rate was low in the <10 mm group. However, both R0 resection and *en-bloc* resection rates were low in the 10–20 mm group, and the AE and residual recurrence rates were not 0%. Although the number of cases was small, both *en-bloc* resection and R0 resection rates were low and the AE rate and residual recurrence rates were high for lesions >20 mm [6,11,20,21,22,23]. If the *en-bloc* resection and R0 resection rates were considered important, ESD would be the better treatment; however, in the duodenum, the AE rate was regarded as more important. Considering the capability to treat residual recurrent lesions after UEMR with re-endoscopic resection, UEMR is currently recommended rather than ESD. A similar result was obtained with cEMR if the lesion size was limited to ≤20 mm. Despite the small number of lesions, the *en-bloc* resection rate was slightly better with cEMR than with UEMR for cases >20 mm; however, the complication rate was almost the same. In contrast to cEMR, it was unclear whether the residual recurrence rate was lower for piecemeal resection in UEMR; conversely, piecemeal resection may also be acceptable.

Previous reports have established the efficacy and safety of UEMR for lesions ≤ 20 mm, and lesions > 20 mm should be avoided when planning *en-bloc* resection. However, it was unclear whether piecemeal UEMR was acceptable and how to distinguish UEMR from cEMR. The advantages of UEMR compared with cEMR are as follows: (1) short time and low labor procedure without submucosal injection, (2) facilitating prophylactic suturing easier due to the soft mucosal defect, and (3) ability to resect lesions with scars. Conversely, the disadvantages were (1) difficulty in creating a water immersion situation, (2) unsecured endoscopic view during snaring, especially on the anal side of the lesions, and (3) risk of aspiration pneumonia. UEMR and cEMR were roughly in the same class of treatment for 11–20 mm lesions, and a direct comparison study between the two needs to be conducted.

### 3.5. Endoscopic Full-Thickness Resection (EFTR)

There have been reports of EFTR as a treatment option for SNADETs that are poorly lifted by submucosal injection due to fibrosis or other factors. In particular, notable outcomes were obtained with an OTSC. Deployment of an OTSC was followed by full-thickness resection, which was performed inside the OTSC by snare. The *en-bloc* resection and R0 resection rates were relatively high (80–100% and 80–92.9%, respectively) without increasing the AE rate; EFTR might be an option when lesions are difficult to resect with ESD if the patient is unable to undergo invasive surgery with general anesthesia [24,25,26].

### 3.6. Laparoscopic and Endoscopic Cooperative Surgery for Duodenal Tumors (D-LECS)

Although LECS was developed for gastric submucosal tumors [27], it has also been adapted for duodenal tumors (D-LECS) [28,29]. D-LECS can be divided into two types depending on whether performing perforation during endoscopic treatment: full-thickness resection (with perforation: D-LECS with FTR) or ESD with reinforcement using a laparoscopic approach (without perforation: D-LECS with ESD). Although perforation is a risk factor for intra-abdominal dissemination of tumor cells, data regarding the best procedure are lacking.

Nunobe et al. [30] reported good treatment outcomes with D-LECS; *en-bloc* resection and R0 resection rates were 96% and 95%, respectively. However, the postoperative perforation of Clavien-Dindo grade ≥ 3 was observed in 1.5% of cases. Notably, even if the mucosal defect is reinforced with seromuscular suturing, the postoperative leak cannot be ignored. Furthermore, there are limited data on the incidence of lymph node dissection in duodenal carcinoma, and it is difficult to clarify whether complete treatment with local excision alone is required in cases of submucosal invasion. However, if endoscopic resection is expected to be difficult owing to a positive preoperative non-lifting sign or advanced fibrosis, we believe that D-LECS could be a treatment option for standard surgery (pancreaticoduodenectomy).

## 4. Conclusions

**Figure 1:**
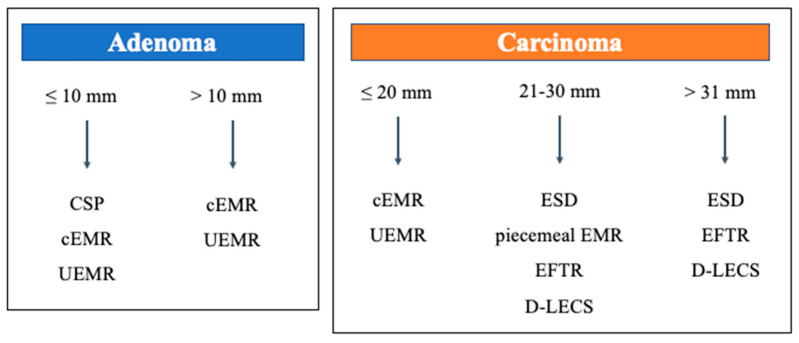
Our current strategy recommendation for SNADETs. cEMR: conventional endoscopic mucosal resection, CSP: cold snare polypectomy, D-LECS: laparoscopic and endoscopic cooperative surgery for duodenal tumors, EFTR: endoscopic full thickness resection, ESD: endoscopic submucosal dissection, SNADET: superficial non-ampullary duodenal epithelial tumors, UEMR: underwater endoscopic mucosal resection.

The current status and challenges of endoscopic treatment for duodenal tumors were discussed based on our results and previous reports. Considering the above results, the current treatment strategy for SNADETs is shown in Figure 1. Here, it is important to note that the present review is related to endoscopic treatment and does not include surgical resection. Additionally, since each institution is in a different situation, with varying endoscopists’ skills and surgical backup systems, and since the heterogeneity of SNADETs needs to be considered, we should be aware that not all patients would benefit from this strategy. Further, although endoscopic resection for SNADETs is not widely accepted, it should be noted that most of the previous reports are published from so-called high-volume centers. Since the indications of CSP, cEMR, and UEMR for adenomas could not be stated in detail, particularly CSP and cEMR—also familiar for colorectal polyps—should be acceptable, including those at non-expert institutions. CSP might be a manageable procedure even for non-experts, considering that the delayed complication is almost 0%. However, severe complications may occur even with CSP; consultation with a high-volume center should be considered if it is difficult to manage such cases [31,32]. Furthermore, several issues need to be solved regarding whether *en-bloc* resection is really required (piecemeal EMR and CSP are acceptable) and whether endoscopic treatment alone is necessary (indications for ESD/EFTR vs. D-LECS). Currently, piecemeal EMR, ESD, EFTR, and D-LECS are described together as treatment options for large lesions (>20 mm); we believe that the treatment strategy will become clearer with the accumulation of further cases and technological development.

## Figures and Tables

**Table 1:** Treatment outcomes of cold snare polypectomy (CSP) at our institution.

	47 Patients/53 Lesions
Age, median (range), years	67 (39–82)
Sex (male/female)	37/10
Location (1st/2nd/3rd)	6/45/2
Size (endoscopic), median (range), mm	6 (2–12)
Macroscopic type (0–I/0–IIa/0–IIa + IIc/0–IIc)	6/43/1/3
Biopsy before CSP	47% (25/53)
Closure after CSP	58% (31/53)
*en-bloc* resection rate	96% (51/53)
Histopathological assessment carcinoma/adenoma/nonneoplastic	3/42/8
R0 resection rate	44% (20/45)
Horizontal margins—negative rate	47% (21/45)
Vertical margins—negative rate	91% (41/45)
Adverse event rate delayed bleeding/intraoperative perforation/delayed perforation	0/0/0
Residual recurrence rate ^a^	2.1% (1/47) ^b^

^a^ One month after CSP. ^b^ Follow-up endoscopy was performed a month after CSP for 47 of the 53 lesions.

**Table 2:** Treatment outcomes of underwater endoscopic mucosal resection (UEMR) at our institution.

	54 Patients/65 Lesions
Age, median (range), years	67 (28–89)
Sex (male/female)	31/23
Location (1st/2nd/3rd)	9/52/4
Size (endoscopic), median (range), mm	12 (3–25)
Macroscopic type (0–I/0–IIa/0–IIa + IIc/0–IIc)	8/36/17/4
Biopsy before UEMR	40% (26/65)
Closure after UEMR	91% (59/65)
*En-bloc* resection rate	86% (56/65)
Histopathological assessment carcinoma/adenoma/nonneoplastic	15/46/4
R0 resection rate	51% (31/61)
Horizontal margins—negative rate	52% (32/61)
Vertical margins—negative rate	97% (59/61)
Adverse event rate delayed bleeding/intraoperative perforation/delayed perforation	1/0/0
Residual recurrence rate ^a^	4.2% (2/48) ^b^

^a^ One month after UEMR. ^b^ Follow-up endoscopy was performed a month after UEMR for 48 of the 65 lesions.

**Table 3:** Summary of UEMR treatment outcomes in previous studies.

	Kiguchi Y, et al. [21]	Iwagami H, et al. [22]	Yamasaki Y, et al. [23]	Yamasaki Y, et al. [20]	Kato M, et al. [6]	Suwa T, et al. [11]
All *En-bloc* R0 HM0 Bleeding Perforation Recurrence	N = 104 87% (90/104) 67% (60/104) 67% (60/104) 2% (2/104) 0% (0/104) -	N = 162 68% (110/162) - 46% (74/162) 1.2% (2/162) 0.6% (1/162) 5% (7/157)	N = 79 77% (61/79) - - 3.8% (3/79) 0% (0/79) 3.8% (3/79)	N = 166 90% (149/166) 67% (111/166) - 1.2% (2/166) 0% (0/166) 2.6% (4/151)	N = 579 79% (455/579) 56% (316/579) - 2.1% (12/579) 0.2% (1/579) -	N = 65 86% (56/65) 51% (31/61) 52% (32/61) 1.5% (1/65) 0% 4% (2/48)
<10 mm *En-bloc* R0 HM0 Bleeding Perforation Recurrence	-		≤10 mm N = 46 96% (44/46) - - 2.2% (1/46) 0% (0/46) 2.2% (1/46)	<10 mm N = 76 100% 75% (57/76) - 0%	< 10 mm 89.1–93.3%	N = 25 96% (24/25) 60% (15/23) 60% (15/23) 0% 0% 0%
10–20 mm *En-bloc* R0 HM0 Bleeding Perforation Recurrence		<20 mm N = 134 79% (106/134) - - - - 2.2% (3/134)	11–20 mm N = 22 73% (16/22) - - 4.5% (1/22) 0% (0/22) 0% (0/22)	≥ 10 mm N = 79 78% (62/79) 58% (46/79) - - 0% -	10–19 mm 62–81.5%	N = 35 89% (31/35) 49% (15/33) 46% (16/33) 0% 0% 3.8% (1/26)
>20 mm *En-bloc* R0 HM0 Bleeding Perforation Recurrence		≥20 mm N = 28 14% (4/28) - - - - 14% (4/28)	>20 mm N = 11 9.1% (1/11) - - 50% (1/2) 0% (0/2) 18% (2/11)		≥20 mm 30%	N = 5 20% (1/5) 20% (1/5) 20% (1/5) 20% (1/5) 0% 20% (1/5)
